# Proteins and Peptides Studied In Silico and In Vivo for the Treatment of Diabetes Mellitus: A Systematic Review

**DOI:** 10.3390/nu16152395

**Published:** 2024-07-24

**Authors:** Isaiane Medeiros, Ana Francisca Teixeira Gomes, Emilly Guedes Oliveira e Silva, Ingrid Wilza Leal Bezerra, Juliana Kelly da Silva Maia, Grasiela Piuvezam, Ana Heloneida de Araújo Morais

**Affiliations:** 1 Biochemistry and Molecular Biology Postgraduate Program, Biosciences Center, Federal University of Rio Grande do Norte, Natal 59078-970, RN, Brazil; isaianemedeirosss@gmail.com; 2 Nutrition Postgraduate Program, Center for Health Sciences, Federal University of Rio Grande do Norte, Natal 59078-900, RN, Brazil; aft.gomes00@gmail.com (A.F.T.G.); e.millyguedes@hotmail.com (E.G.O.e.S.);; 3 Nutrition Department, Center for Health Sciences, Federal University of Rio Grande do Norte, Natal 59078-900, RN, Brazil; 4 Public Health Department, Federal University of Rio Grande do Norte, Natal 59078-900, RN, Brazil; 5 Health Sciences Postgraduate Program, Health Sciences Center, Federal University of Rio Grande do Norte, Natal 59078-900, RN, Brazil

**Keywords:** hyperglycemia, therapy, computer simulation, animals

## Abstract

Bioinformatics has expedited the screening of new efficient therapeutic agents for diseases such as diabetes mellitus (DM). The objective of this systematic review (SR) was to understand naturally occurring proteins and peptides studied in silico and subsequently reevaluated in vivo for treating DM, guided by the question: which peptides or proteins have been studied in silico for the treatment of diabetes mellitus? The RS protocol was registered in the International Prospective Register of Systematic Reviews database. Articles meeting the eligibility criteria were selected from the PubMed, ScienceDirect, Scopus, Web of Science, Virtual Health Library (VHL), and EMBASE databases. Five studies that investigated peptides or proteins analyzed in silico and in vivo were selected. Risk of bias assessment was conducted using the adapted Strengthening the Reporting of Empirical Simulation Studies (STRESS) tool. A diverse range of assessed proteins and/or peptides that had a natural origin were investigated in silico and corresponding in vivo reevaluation demonstrated reductions in glycemia and/or insulin, morphological enhancements in pancreatic β cells, and alterations in the gene expression of markers associated with DM. The in silico studies outlined offer crucial insights into therapeutic strategies for DM, along with promising leads for screening novel therapeutic agents in future trials.

## 1. Introduction

Bioinformatics plays a pivotal role in various fields of study, particularly in the healthcare sector, where it has been applied in the development of new natural therapeutic agents and/or medications against various diseases [1]. Research indicates that in silico studies are key elements for the identification, enrichment, and preliminary validation of therapeutic targets. Moreover, these studies serve as a strategy for developing more effective natural and synthetic biomarkers, investigating and mitigating toxicity, and enhancing pharmacogenomics [1,2].

In this context, in silico studies using computer-aided drug design (CADD) tools, which encompass structure-based drug design (SBDD) and ligand-based drug design (LBDD) approaches [3], have assisted in the identification and development of new drugs.

Therefore, in silico studies anticipate structures, sequences, and bioactive actions, facilitating the identification of active pharmacophores and optimizing coupling mechanisms. Consequently, they enable the assessment of the effectiveness of action and/or inhibition modes on the analyzed therapeutic targets [3,4]. These possibilities are made possible through virtual screening techniques, such as molecular docking or molecular dynamics simulations, which guide the selection of proteins or genes for the development of new drugs by considering their respective targets [1].

Thus, in silico drug design has expedited the fast screening of efficient compounds or biomolecules for pre-clinical and clinical research [1], leading to the development of therapeutic agents for diseases such as obesity, hypertension, and diabetes mellitus.

Diabetes mellitus (DM) is a public health issue that affected approximately 537 million people worldwide in 2021, and projections indicate a rise to 783 million by 2045 [5]. This disease arises from metabolic disorders in carbohydrate metabolism, resulting in hyperglycemia due to glucose underutilization as an energy source and overproduction through gluconeogenesis and glycogenolysis [6].

The treatment of DM involves medications that block or inhibit therapeutic targets, such as dipeptidyl peptidase IV (DPP-IV), insulin receptor (IR), α-glucosidase, α-amylase, glucose transporter type 2 (GLUT2), and sodium-glucose transport protein (SGLT-1) [7]. Some drugs, like metformin, sitagliptin, vildagliptin, and saxagliptin, have been utilized for DM treatment based on their action on these targets. However, these commercially available medications often lead to adverse side effects, including hypoglycemia, headaches, dizziness, lactic acidosis, gastrointestinal disorders, weight gain, peripheral edema, renal insufficiency, and diarrhea [8,9,10]. These effects motivate the need for new molecules for the treatment of DM, particularly proteins and peptides.

Indeed, in recent years, peptide drugs have garnered attention for their potential benefits in treating diseases such as DM, given their increased potency, tissue-specificity, and minimal side effects [11,12]. According to De La Torre and Albericio [13], between 2015 and 2019, the United States Food and Drug Administration (FDA) approved 15 peptide drugs, constituting 7% of the total drugs approved during this period. Furthermore, recent advances in peptide screening and computational biology approaches have facilitated the development of new peptide drugs [11]. However, there remain criticisms regarding computational studies when used as the primary decision-making strategy, and therefore, many studies were developed in silico and reproduced in vivo in an attempt to validate computational findings.

Despite these criticisms, bioinformatics tools have been extensively employed for screening compounds and/or active molecules, including proteins and peptides. Thus, the objective of this review was to investigate the natural proteins and/or peptides studied for the benefit of DM treatment, analyzed in silico, and subsequently evaluated in vivo. The guiding question for this review was as follows: which peptides or proteins have been studied in silico for the treatment of diabetes mellitus?

## 2. Methodology

This systematic review was based on a protocol developed following the guidelines described in the Preferred Reporting Items for Systematic Review and Meta-Analysis Protocols (PRISMA-P) [14] (Table S1) and registered in the International Prospective Register of Systematic Reviews (PROSPERO) on 3 September 2022 (CRD42022355540) [15,16].

The initial study proposal considered the inclusion of publications without delimitation of the type of therapeutic agent evaluated, as long as it was conducted with proteins or peptides, as outlined in the systematic review protocol [15]. However, upon initiating the literature searches, it became evident that there was a necessity to restrict these agents to those derived from natural sources. This decision arose from the existence of studies involving various synthetic proteins and peptides, whose distinctions could potentially complicate the analysis and discussion of the data. Consequently, adjustments were made and registered in PROSPERO [15].

Three independent researchers directly participated in all phases of this systematic review, with discrepancies resolved through discussion with a fourth reviewer [15].

### 2.1. Search Strategies

Initially, search equations were tested using keywords indexed in the Medical Subject Heading (MESH), and equations with different combinations (Table 1) were formulated to identify natural peptides/proteins with potential applications in the treatment of DM. The search was independently conducted by two researchers (I.M. and A.F.T.G.) in February 2024 in the following databases: PubMed; ScienceDirect; Scopus; Web of Science; Virtual Health Library (BVS); and EMBASE.

The articles were searched through access to the “Portal Periódicos Capes” in Brazil, associated with CAFE access using the UFRN—Universidade Federal do Rio Grande do Norte system login, which allowed access to all studies included in this review.

### 2.2. Elegibility Criteria

#### 2.2.1. Inclusion Criteria

Peer-reviewed original journal articles meeting the eligibility criteria based on population, exposure, and context (PECo) (Table 2) were included in the review, with no language restriction. Additionally, only studies presenting both in silico and in vivo analyses involving the same peptides or proteins demonstrating their effects on the diabetic condition were included.

#### 2.2.2. Exclusion Criteria

Exclusively in vivo or in vitro studies were excluded, as well as preprints, review articles, theses, dissertations, letters, case reports, editorials, conference abstracts, grey literature, and molecular dynamics studies focused on other comorbidities.

### 2.3. Data Extraction

The studies were selected based on the search strategy and the aforementioned databases (Table 3). Following classification, all articles were imported into the Rayyan app (version 0.1.0). This platform facilitated the removal of duplicate studies and allowed for the inclusion of titles and abstracts that met the study’s inclusion and exclusion criteria.

Three reviewers independently extracted their respective data: authors, year of publication, origin of proteins and/or peptides, model (in silico), technique employed (docking or molecular dynamics), docking score or potential interaction energy (PIE), assessments conducted, and outcome (effect of therapeutic agents on fasting blood glucose, oral glucose tolerance test, insulin, insulin expression, etc.). Correspondence was initiated via email (with at least two attempts) with corresponding authors to obtain full papers for evaluation if not available initially. These data were tabulated in a predefined table using Microsoft Excel.

### 2.4. Assessment of Risk of Bias and Study Quality

The quality of the modeling was assessed using a checklist developed based on standardized guidelines for simulation “Strengthening the Reporting of Empirical Simulation Studies (STRESS)” (adapted). As no standardized tool exists specifically for this type of study, the bias risk was evaluated based on a checklist obtained from different literature sources, such as the checklist developed by Taldaev et al. [22], with a slight adaptation for subdividing criterion 13 into in vitro and in vivo assessment [23]. The assessment of the study quality was conducted independently, following prior training to ensure consistency in the application of the tool.

## 3. Results

### 3.1. Selection and Characteristics of Studies

**Figure 1:**
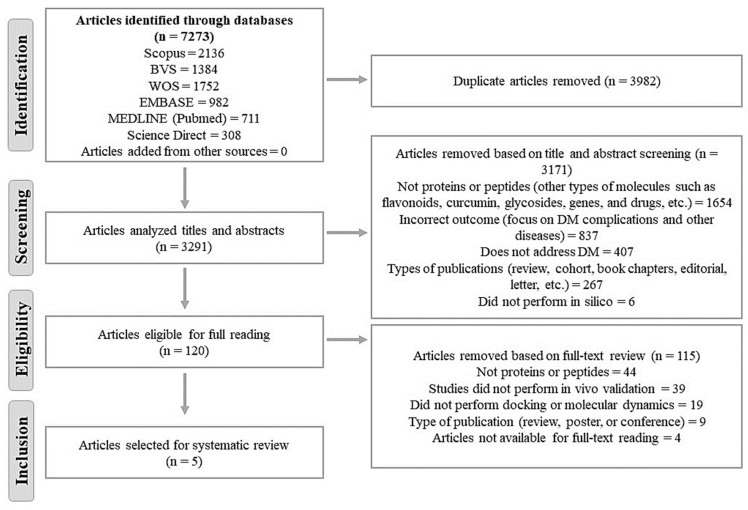
Flowchart of article selection steps for systematic review. Adapted from PRISM [24]. PRISM: Preferred Reporting Items for Systematic Reviews and Meta-Analysis Protocols. BVS: Virtual Health Library. WOS: Web of Science. EMBASE: Excerpta Medica Database. DM: diabetes mellitus.

A total of 7273 articles were initially identified, with 3982 articles removed as duplicates. Out of the 3291 studies subjected to title and abstract screening (the initial screening phase), 120 articles were selected for full-text reading, of which 5 articles were ultimately included in this systematic review (SR). During the initial screening phase, 52.2% of articles (*n* = 1654) were excluded because they did not involve studies with proteins and/or peptides; 26.4% (*n* = 837) focused on diabetic neuropathies, cancers, and Alzheimer’s, among other diseases or diabetic complications, rather than on DM itself; 12.8% (*n* = 407) did not discuss DM; 8.4% (*n* = 267) were not original articles, and 0.2% (*n* = 6) did not involve in silico studies. In the final full-text reading phase, 38.3% of articles (*n* = 44) were excluded for not presenting evaluations with therapeutic agents of interest (proteins and/or peptides of natural origin), 33.9% of studies (*n* = 39) did not conduct in vivo assays with the same peptides for validation of the data obtained in silico, 16.5% (*n* = 19) did not conduct docking or molecular dynamics, 7.8% (*n* = 9) were not original articles, and 3.5% (*n* = 4) were not found or made available through contact with corresponding authors (Figure 1).

The studies selected for this SR featured natural proteins and/or peptides, along with in silico and in vivo outcomes related to DM evaluation. The studies displayed a diverse range in the amino acid structures of the therapeutic agents under evaluation. Concerning the origins of the biomolecules, investigations were conducted with peptides or proteins of plant origin (n = 2), animal origin (n = 2), and both origins (n = 1), targeting in silico therapeutic targets, such as dipeptidyl peptidase IV (DPP-IV) (n = 1), insulin receptor (n = 2), α-glucosidase (n = 1), glucose transporter type 2 (GLUT2), and sodium-glucose transport protein (SGLT-1) (n = 1). All studies utilized molecular docking as an in silico technique; however, among these, only one study applied molecular dynamics in conjunction with molecular docking (Table 3).

### 3.2. Quality of Studies

**Figure 2:**
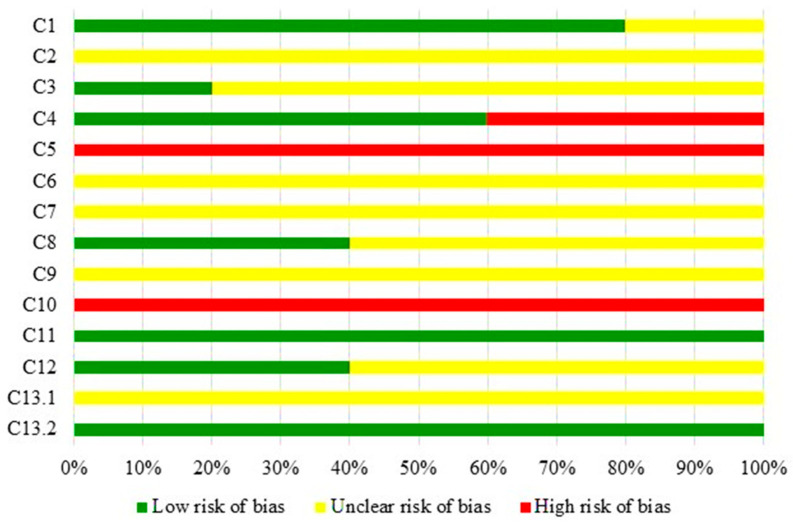
Assessment of the risk of bias of the studies included in this systematic review, according to the adaptation made in the evaluation tool developed by Taldaev et al. [22,23].

The risk of bias assessment for the studies included in this SR adhered to the adapted [23] checklist developed by Taldaev et al. [22] (Figure 2). The majority of articles did not provide sufficient reporting on the criteria evaluated in this checklist, resulting in an unclear risk of bias (as indicated by the yellow shading in Figure 2).

In terms of criterion 1 (C1), pertaining to ligand selection, 80% of the studies described how ligand selection was conducted. Meanwhile, all the studies (100%) did not clearly evaluate ionization due to the absence of pKa and pH data of the medium (C2), and 80% did not address the ligand potential energy calculation (C3).

Regarding target selection, it was observed that 60% of the studies utilized targets with resolution values up to 2.5 Å (C4), and 100% presented structures generated by X-ray crystallography (C5). Concerning criteria 6 to 9 (C6 to C9), which pertain to target optimization, the majority of studies exhibited an uncertain risk of bias assessment by not reporting the control of histidine protonation (100%), amino acid protonation after X-ray crystallography (100%), the addition of missing residues and side chains after X-ray crystallography (60%), and the addition of metals (100%).

The software employed for molecular docking showed a high risk of bias (100%) due to not utilizing software such as GOLD or Glide (C10). Lastly, regarding the last three criteria (C11, C12, and C13) related to the result evaluation, it was observed that 100% of the studies conducted visual inspection. However, 60% of the studies did not assess re-docking, and 100% lacked comprehensive data evaluation of in vitro studies. Among these, 60% of studies (three studies) conducted and presented quantitative calculations but did not provide a binding constant (C13.1), and 100% of the studies carried out an in vivo validation (C13.2).

## 4. Bioactive Proteins and Peptides Evaluated In Silico and In Vivo for the Treatment of DM

From the studies selected to comprise this SR (Table 3), heterogeneity was observed among the selected proteins and/or peptides, both in terms of their origins and amino acid compositions. When assessing the bias risk of the included articles, it was noted that most criteria were classified as having an uncertain bias risk due to the lack of detailed methodologies regarding the in silico analyses. However, two criteria (C5 and C10) showed a high risk of bias, as all included articles referenced the therapeutic target structure from the RCSB Protein Data Bank (PDB) without using software recognized as gold standards in docking and molecular dynamics assessment (GOLD and GLIDE). The choice of software is essential in assessing the interaction between therapeutic agents and their target molecules. There are several software programs or servers available for docking or molecular dynamics; however, not all of them have sufficient sensitivity to evaluate such interactions. Taldaev et al. [22] recommends Glide and GOLD as the gold standard for molecular docking studies. Furthermore, studies focusing on nutraceuticals have indicated that among the software options available, AutoDock Vina, Glide, and AutoDock GOLD are the most recommended choices for docking and molecular dynamics evaluation with the highest scores [25,26,27]. However, the software differs in terms of their algorithm methods and scoring used. Glide and GOLD, for instance, employ screening with a Monte Carlo algorithm and empirical scoring, thus demonstrating superior results [22].

Additionally, certain steps are crucial for conducting studies involving a bioinformatics approach and assessing bias risk in tools. The selection of ligands, which was the focal point of this study, stands as one of the initial stages guiding the discovery of potential therapeutic agents. Common errors in this phase include the neglect to filter ligands’ three-dimensional structures or the use of low-reliability software for molecule construction [28]. To address these issues, the utilization of three-dimensional screening libraries, such as PubChem, ZINC, and ChemSpider, is recommended, as they provide comprehensive information on various ligands, aiding in proper filtration [22,29]. Additionally, software like Modeller version 10.5, http://www.salilab.org/modeller/ (accessed on 14 June 2024), I-Tasser version 5.1, https://zhanglab.ccmb.med.umich.edu/I-TASSER/ (accessed on 14 June 2024), and Swiss-model, http://swissmodel.expasy.org/ (accessed on 14 June 2024), facilitate model construction through homology modeling, deriving models from structurally highly homologous sequences, which is crucial in the absence of three-dimensional structures [30].

Another important step involves selecting the therapeutic target while considering specific characteristics, such as the target structure resolution and the method of acquisition. Taldaev et al. [22] recommended that the structure should have a resolution of up to 2.5 Å, with nuclear magnetic resonance (NMR) spectroscopy being the preferred method of acquisition. However, Pantsar and Poso [31] emphasized that X-ray crystallography is the most common tool, despite potentially providing partial information with low-quality resolution and electron density. Nevertheless, it remains a promising experimental method for elucidating biomolecules [32].

Finally, common errors in bioinformatics studies often arise due to the absence of visual verification of identified interactions, lack of re-docking, and failure to validate these interactions through in vitro or in vivo studies [1]. Methods such as refinement and reevaluation of re-docking are crucial in the quest for therapeutic agents, as they enhance the accuracy rates in virtual screening campaigns and improve the correlation with experimental data [28,33,34]. For this purpose, it is strongly recommended to include figures depicting observed interactions to facilitate readers’ visualization, conduct re-docking to validate the appropriateness of parameters used for the evaluated systems, and perform in vitro or in vivo validation to affirm the results of theoretical modeling [29,35].

Thus, this SR suggests that studies conducted with computational analyses should use checklists, such as the one developed by Taldaev et al. [22], even if adapted, in order to enhance the methodological description, improve method detailing, and, consequently, present a higher quality. Therefore, Taldaev et al. [22] were pioneers in assessing the bias risk in silico studies and also addressed limitations, such as protein target processing and molecular docking evaluation.

The five selected studies addressed the guiding question (which peptides or proteins have been studied in silico for the treatment of diabetes mellitus?), met the pre-established criteria outlined in the methodology, and were conducted using proteins and/or peptides of natural origin (animal or plant) [17,18,19,20,21].

Wan et al. [17] investigated the potential antidiabetic effects of protein hydrolysates obtained by enzymatic hydrolysis (trypsin) of *T. ovatus* fish muscles (TOH) in streptozotocin-induced diabetic mice (male Kunming mice). Carnivorous marine fish are commonly found in the temperate and subtropical waters of the Pacific, Indian, and Atlantic Oceans [36]. The authors observed a significant reduction in fasting blood glucose, which was dose-dependent (16% for 100 mg/kg of TOH, 21% for 500 mg/kg of TOH, and 27% for 1000 mg/kg of TOH). Additionally, there was an increase in plasma insulin concentrations in the groups treated with TOH and metformin compared with the untreated diabetic control group. Furthermore, improvement in the regular contour morphology of pancreatic islets and recovery of the number of cells in these islets was observed after treatment with TOH and metformin.

In the same study, the active peptide sequences of the protein hydrolysates were identified and traced by HPLC-ESI-Q-TOF-MS/MS combined with bioinformatics tools, and the possible inhibition mechanisms for α-amylase (AAM) (PDB ID 3BAJ, resolution 2.10 Å) and DPP-IV (PDB ID 4A5S, resolution 1.62 Å) of these peptides were preliminarily analyzed by molecular docking using the software Discovery Studio version 4.5. It was observed that the peptide FNFSR (Phenylalanine-Asparagine-Phenylalanine-Serine-Arginine) (with an arginine residue at the *C*-terminal) bound to diabetic targets (AAM and DPP-IV) via non-bonding interactions (hydrogen bonds, van der Waals forces, and π–π interactions). These interactions occurred at the active sites of the targets. For DPP-IV, the active site comprises a cavernous site consisting of S1 (catalytic triad—Serine 630, Aspartate 708/Asparagine 710, and Histidine 740—and hydrophobic residues—Tyrosine 547, Tyrosine 631, Tryptophan 659, Tyrosine 662, Tyrosine 666, Valine 711, and Valine 656) and S2 (Glutamate 205, Glutamate 206, and Arginine 125). Regarding AAM, it comprises three domains (A, B, and C), featuring a catalytic triad (Aspartate 300, Glutamate 233, and Aspartate 300) and allosteric sites (Aspartate 96, Arginine 195, Histidine 15, Glutamine 41, Valine 42, Serine 43, Proline 44, and Arginine 337), which are targeted by the bioactive peptides present in this study [17].

Guru et al. [18] identified peptides from a transcriptome database (constructed from protein sequences) derived from the amino acid sequence of peptides from a teleost Channa striatus, which is a popular food fish in Southeast Asia. They assessed the impact of the peptides on pancreatic β-cells, 2NBDG (2-deoxy-2-((7-nitro-2,1,3-benzoxadiazol-4-yl)amino)) uptake, plasma glucose levels, insulin gene expression, and transcriptional expression of phosphoenolpyruvate carboxykinase (PEPCK) in an experimental study with diabetic zebrafish (Danio rerio, both sexes) induced by alloxan. The authors found that the use of the peptide WL15 (WHKNCFRCAKCGKSL: Tryptophan, Histidine, Lysine, Asparagine, Cysteine, Phenylalanine, Arginine, Cysteine, Alanine, Lysine, Cysteine, Glycine, Lysine, Serine, Leucine) exhibited a protective effect against damage to pancreatic β-cells and the increase in the size of these cells in larvae. Additionally, there was a significant reduction in the plasma glucose concentration, positive regulation of insulin gene expression, and inhibition of PEPCK mRNA expression.

Furthermore, several peptides were identified, and WL15, derived from the protein sequence 2, which is rich in cysteine and glycine (CSRP2), was selected for further in vivo study. This peptide showed higher binding affinity to the insulin receptor (in silico) (PDB ID 1IR3, resolution 1.90 Å) by molecular docking using the software Discovery Studio version 4.1, greater α-amylase inhibition (in vitro), and prevention of morphological abnormalities in zebrafish in a dose-dependent manner (in vivo). The amino acids demonstrating the highest interaction between WL15 and the insulin receptor were Histidine 1081, Aspartate 1083, Histidine 0, Tryptophan 1, Lysine 3, Leucine 1002, Glutamate 1004, Serine 1066, Glutamate 1043, Glutamate 1040, Leucine 15, Lysine 13, Glycine 12, Glutamate 1216, and Alanine 9 [18].

Costa et al. [19] evaluated the effects of tamarind seed trypsin inhibitor (TTI) derived from *Tamarindus indica*, which is a plant native to Africa and cultivated in humid and arid tropical climates, on fasting blood glucose, insulinemia, insulin resistance indices, pancreatic β-cell functionality, and insulin sensitivity in male Wistar rats with type 2 diabetes induced by a high glycemic index and high glycemic load diet. Additionally, they conducted computational studies involving molecular docking and molecular dynamics using a theoretical model of TTI number 56 and conformation number 287 (TTIp 56/287) to evaluate the interaction of this protein with the insulin receptor. They observed a reduction in the plasma glucose and HOMA-IR and an increase in HOMA-β, but no alteration in the plasma insulin levels when compared with untreated diabetic animals.

Furthermore, TTIp 56/287 interacted with the isoform B of the insulin receptor (PDB ID 4OGA, resolution 3.50 Å) by molecular docking using the online server Molecular Docking Algorithm Based on Shape Complementarity Principles (Patchdock) and molecular dynamic using the GROningen Machine for Chemical Simulations (GROMACS) version 2018.4, which is a region specific for promoting insulin-mediated signal transduction, as well as with insulin, albeit in a region adjacent to it. This study proposed that TTI could be an insulin mimetic molecule because its theoretical version bound to a different region than that occupied by insulin on the insulin receptor. Therefore, it could activate or modulate signaling pathways that promote glycemic control. TTIp 56/287, consisting of 70 amino acid residues, interacts with the insulin receptor through the residues of Aspartate 7, Glutamine 47, Aspartate 145, Arginine 59, and Serine 34 [19].

Huang et al. [20] observed the in vitro inhibition of α-glucosidase, α-amylase, and DPP-IV using the hydrolysates obtained from Corbicula flumineas (ACH), which is a freshwater clam (Asian clam) and a functional food in Asia, through hydrolysis, and from subtilisin (protamex) and Chlorella sorokiniana (PCH), which is a nutrient-rich green algae, through digestion with cellulose AP3 and protease N. The peptides were purified, sequenced, and administered to male diabetic rats (Sprague Dawley) induced by nicotinamide and streptozotocin through single or combined supplementation of ACH and PCH. ACH yielded two peptides (VKP: Valine, Lysine, Proline and VKK: Valine, Lysine, Lysine), and from PCH, four peptides were obtained (VW: Valine, Tryptophan; WV: Tryptophan, Valine; IW: Isoleucine, Tryptophan; and LW: Leucine, Tryptophan). It was found that ACH significantly inhibited all analyzed therapeutic targets, whereas PCH did not inhibit α-amylase. When the hydrolysates were combined (ACH/PCH), enhanced results were observed at ratios of 1:3 and 1:1, along with a significant reduction in blood glucose after 30 min of ingestion, modulation of insulin resistance, and improvement in insulin sensitivity.

Regarding the in silico analysis by molecular docking using pepATTRACT software, http://bioserv.rpbs.univ-paris-diderot.fr/%20services/pepATTRACT/ (accessed on 14 June 2024), and visualized using the Swiss PDB Viewer version 4.1, https://spdbv.unil.ch/ (accessed on 14 June 2024) peptides from ACH interacted with the catalytic sites D518 and D616 of α-glucosidase (PDB ID 5KZW, resolution 2.0 Å), thereby inhibiting its activity. Conversely, peptides from PCH interacted with the β-chains of the catalytic domain GH31, leading to inhibitory action. Regarding DPP-IV, peptides from ACH and PCH were situated in the α/β-hydrolase domain of DPP-IV, which contains three catalytic residues (Serine 630, Aspartate 708, Histidine 740), along with a β-helix domain with two substrate-anchoring residues (Glutamate 205 and Glutamate 206) [20].

Mojica et al. [21] evaluated the hypoglycemic potential of a hydrolyzed protein isolate (HPI) derived from black beans (Black Otomi) and its individual peptides (AKSPLF: Alanine, Lysine, Serine, Proline, Leucine, Phenylalanine; ATNPLF: Alanine, Threonine, Asparagine, Proline, Lysine, Phenylalanine; FEELN: Phenylalanine, Glutamate, Glutamate, Leucine, Asparagine; LSVSVL: Leucine, Serine, Valine, Serine, Valine, Leucine), using in silico, in vitro, and in vivo methods. Protein hydrolysis in the beans was achieved using the Alcalase enzyme. In vitro assays were conducted with Caco-2 cells (human colon epithelial cells) with glucose addition to simulate the development of DM. In the in vitro analysis, pure peptides and HPI were administered as treatments, resulting in a significant reduction in glucose absorption. The reductions ranged from 8.5% for the AKSPLF peptide (100 μM) to 21.5% for HPI (10 mg/mL), accompanied by a decreased glucose uptake. The LSVSVL peptide showed the most potent inhibitory effect on the glucose uptake.

According to Mojica et al. [21], computational modeling revealed that the peptides AKSPLF, ATNPLF, FEELN, and LSKSVL could block the glucose transporters, GLUT2 and SGLT1 (PDB ID 3DH4, resolution 2.70 Å), by interacting with their protein sites. The interaction of the peptides with GLUT2 (P12336, UniProtKB database, homology modeling) was mainly through hydrophobic interactions, followed by polar interactions visualized by molecular docking using the software DockingServer, http://www.dockingserver.com (accessed on 14 June 2024), and AutoDock tools version 3.0. The FEELN peptide showed the lowest in silico inhibition constant (1.5 μM) for GLUT2. Conversely, the peptides exhibited better binding to SGLT1, with the interactions primarily being polar, followed by hydrophobic interactions. The AKSPLF peptide showed the lowest in silico inhibition constant (Ki) for SGLT1 (0.452 μM).

Regarding the assays with streptozotocin-induced diabetic and normoglycemic male Wistar rats treated with HPI at varying dosages, it was found that the glycemic response was dose-dependent. Additionally, the authors observed that the groups treated with HPI and insulin showed a significant reduction in blood glucose levels, while those treated with metformin and glibenclamide did not perform as effectively in reducing hyperglycemia at the doses administered compared with untreated diabetic animals. Finally, normoglycemic animals treated with different doses of HPI showed no statistical differences in their plasma glucose levels compared with the non-diabetic control group, despite the reduced value compared with normoglycemic animals treated with medications [21].

**Figure 3:**
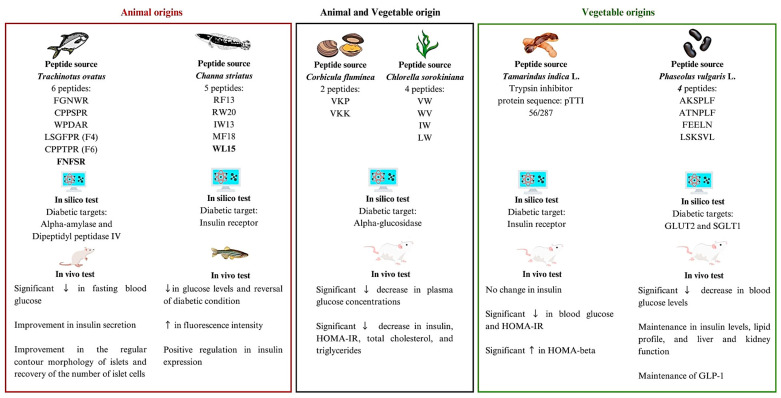
Summary scheme of articles found in this systematic review. According to Mojica et al. [21], A: Ala (Alanine); C: Cys (Cysteine); D: Asp (Aspartate or Aspartic Acid); E: Glu (Glutamate or Glutamic Acid); F: Phe (Phenylalanine); G: Gly (Glycine); I: Ile (Isoleucine); K: Lys (Lysine); L: Leu (Leucine); M: Met (Methionine); N: Asn (Asparagine); P: Pro (Proline); R: Arg (Arginine); S: Ser (Serine); T: Thr (Threonine); V: Val (Valine); W: Trp (Tryptophan); IR: insulin receptor. GLUT2: glucose transporter type 2. SGLT1: sodium-glucose transport protein. GLP-1: Glucagon-like peptide-1. ↑ increase, ↓ decrease.

The studies included in this systematic review (SR) demonstrate that the selected peptides and/or proteins, despite their heterogeneous origins, target the catalytic sites of therapeutic DM targets (Figure 3). Additionally, they evidenced in vivo improvements in biochemical parameters, gene expression, and morphology related to DM.

One limitation of the studies included in this SR was the lack of detailed in silico analyses, hindering the reproducibility and complicating the retrieval of results. Due to the absence of a consolidated and validated protocol for writing in silico articles, it is recommended, based on the findings of this review, to provide a more comprehensive description of the methodological techniques used in computational analyses. This would result in improvements in the methodological quality and facilitate a broader scope for comparison between the models employed in these studies. Therefore, the use of tools such as the one proposed by Taldaev et al. [22] provides methodological rigor to all important steps for the successful execution of bioinformatics studies. Moreover, computational studies employed peptides as therapeutic agents to assess interactions with their targets, contrasting with in vivo investigation that utilized protein hydrolysates derived from natural sources (animals and/or plants) in animal models. This raises the possibility of bias, as the effects observed could potentially be attributed to other molecules inherent to the hydrolysates. Additionally, considering their potential as therapeutic agents treating diabetes mellitus, it is important to evaluate the risk of toxicity and allergenicity of these peptides. This can be explored using in silico tools, complemented by in vitro and in vivo studies, which can elucidate the safety profile of the proposed agents. Nevertheless, this study contributes to advancing the quest for potential therapeutic agents of natural origin with reduced adverse effects, which holds promise for future applications in clinical studies.

## 5. Conclusions

The results obtained in this SR demonstrate the heterogeneity of the origin and amino acid composition of proteins and/or peptides, highlighting those of animal origin (fish and mollusks) and vegetable origin (green microalgae, tamarind seeds, and black beans), regarding their efficacy on various therapeutic targets (DPP-IV, AAM, RI, GLUT2, and SGLT1) of DM. However, per this SR, in silico studies can be considered strategic in the search or screening of new therapeutic agents, considering that all those agents analyzed in silico, included in this SR, were also reassessed in vivo and maintained the consistent potential for acting in the treatment of DM. Therefore, they serve to guide future research, particularly clinical trials, and aid in the quest for novel treatments for this disease.

## Figures and Tables

**Table 1:** Search strategy equations used in the databases to select articles for this systematic review, guided by the research question: “Which peptides or proteins have been studied in silico for the treatment of diabetes mellitus?”.

Database	Search Strategies
PubMed, Scopus, Science Direct, and BVS	(Protein OR peptides) AND (“in silico” OR “computer simulation”) AND diabetes
WOF ^1^ and EMBASE	(Protein OR peptides) AND (“in silico” OR “computer simulation”) AND diabetes

^1^ No filtering was performed in this database. Other databases were filtered by title, abstract, and subject. BVS: Virtual Health Library. WOF: Web of Science. EMBASE: Excerpta Medica Database.

**Table 2:** Elements of the research question according to the PECo strategy for systematic reviews.

Description	Abbreviation	Elements of the Question
Population	P	Proteins or peptides of natural origin
Exhibition	E	Diabetes *mellitus*
Context	Co	In silico studies with molecular dynamics or molecular docking

**Table 3:** Characteristics of studies included in this systematic review.

References	Origin of Proteins and/or Peptides	Proteins and/or Peptides	Techniques Employed	*Docking* Score or Potential Interaction Energy (PIE) with Target of Interest	In Vivo Assessments (Validation)	Outcomes
Wan et al. (2023) [17]	Carnivorous marine fish (*Trachinotus ovatus*)	Protein hydrolysates from carnivorous marine fish (TOH)—6 peptides: FGNWR, CPPSPR, FNFSR, WPDAR, LSGFPR (F4), and CPPTPR (F6)	Molecular docking	Not reported	Fasting blood glucose, insulin, renal histopathology, and pancreatic histopathology	Significant ↓ in fasting blood glucose ^1^ Significant ↑ in fasting blood glucose ^2,3^ - Improvement in insulin secretion, but associated with loss of pancreatic β cells - Improvement in the regular contour morphology of islets and recovery of the number of islet cells after treatment with dose-dependent TOH and metformin
Guru et al. (2022) [18]	Teleost fish (*Channa striatus*)	5 Teleost fish peptides extracted using PeptideRanker: WL15 (WHKNCFRCAKCGKSL), RF13 (RRGKGGRRVTMSF), RW20 (RPVKRKKGWPKGVKRGPPKW), IW13 (IKHFKKQRRLIPW), and MF18 (MRKKAVKVKHVKRREKKF)	Molecular docking	IR: WL15 = −218.16 kcal/mol RF13 = −189.35 kcal/mol RW20 = −191.54 kcal/mol IW13 = −186.32 kcal/mol MF18 = −177.15 kcal/mol	2NBDG uptake, glucose estimation in larvae, and gene expression of insulin and PEPCK	↑ in fluorescence intensity with co-treatment of WL15 (50 µM) ↓ in glucose levels and reversal of diabetic condition with co-treatment of WL15 (50 μM) - Positive regulation in insulin expression when co-treated with WL15 (50 µM)
Costa et al. (2022) [19]	Tamarind seed (*Tamarindus indica* L.)	Trypsin inhibitor protein sequence: pTTI 56/287	Docking and molecular dynamics	IR: TTIp 56/287: −100.6 kJ mol^−1^	Fasting glycemia, insulinemia, HOMA-IR, and HOMA-beta	- No change in insulin Significant ↓ in blood glucose and HOMA-IR ^1^ Significant ↑ in HOMA-beta ^1^
Huang et al. (2022) [20]	The Asian clam (*Corbicula flumínea*) and green microalgae (*Chlorella sorokiniana*)	*Corbicula fluminea* protein hydrolyzate (ACH)—2 peptides: VKP and VKK *Chlorella sorokiniana* protein hydrolyzate (PCH)—4 peptides: VW, WV, IW, and LW	Molecular docking	Not reported	Fasting blood glucose, oral glucose tolerance test, insulin, HOMA-IR, total cholesterol, and triglycerides	Significant ↓ in decrease in plasma glucose concentrations in diabetic animals treated with ACH (9.5%), PCH (4.1%), or ACH/PCH (14.6%) Significant ↓ in plasma glucose concentration at 30 min in diabetic rats fed ACH/PCH in OGTT Significant ↓ in insulin, HOMA-IR, total cholesterol, and triglycerides ^1^
Mojica et al. (2017) [21]	Black bean (*Phaseolus vulgaris* L.)	Black bean protein hydrolyzate (HPI)—4 peptides: AKSPLF, ATNPLF, FEELN, and LSKSVL	Molecular docking	GLUT2: AKSPLF: −0.6 kcal/mol ATNPLF: −7.5 kcal/mol FEELN: −7.91 kcal/mol LSKSVL: −5.24 kcal/mol SGLT1: AKSPLF: −8.66 kcal/mol ATNPLF: −7.20 kcal/mol FEELN: −6.44 kcal/mol LSKSVL: −4.4 kcal/mol	Oral glucose tolerance test Determination of glucose, lipid profile, and renal function Insulin and GLP-1	Significant ↓ in blood glucose levels (22.7 to 47.7%) ^1^ - Maintenance in insulin levels, lipid profile, liver, and kidney function ^1^ - Maintenance of GLP-1 ^1,2^ (except for diabetic treated with HPI 150 mg/kg, where there was a significant increase)

Legend: ↑ increase, ↓ decrease. According to Mojica et al. [21], A: Ala (Alanine); B: Asx (Asparagine or Aspartate); C: Cys (Cysteine); D: Asp (Aspartate or Aspartic Acid); E: Glu (Glutamate or Glutamic Acid); F: Phe (Phenylalanine); G: Gly (Glycine); H: His (Histidine); I: Ile (Isoleucine); K: Lys (Lysine); L: Leu (Leucine); M: Met (Methionine); N: Asn (Asparagine); P: Pro (Proline); Q: Gln (Glutamine); R: Arg (Arginine); S: Ser (Serine); T: Thr (Threonine); V: Val (Valine); W: Trp (Tryptophan). IR: insulin receptor. GLUT2: glucose transporter type 2. SGLT1: sodium-glucose transport protein. 2NBDG: (2-(N-(7-Nitrobenz-2-oxa-1,3-diazol-4-yl)Amino)-2-Deoxyglucose). PEPCK: Phosphoenolpyruvate carboxykinase. GLP-1: Glucagon-like peptide-1. ^1^: These data are related to the test group compared with the diabetic control group. ^2^: These data are related to the test group compared with the normoglycemic control group. ^3^: These data are related to the test group compared with the group treated with metformin. The Shapiro–Wilk test was used to assess the normality of the data. For non-parametric data, the Mann–Whitney or Kruskal–Wallis test followed by Dunn’s post hoc test were used. For parametric data, one-way ANOVA followed by Duncan’s multiple range test or Tukey’s post hoc test was used. The results were considered to be statistically significant at *p* < 0.05.

## Data Availability

Data are contained within the article.

## Supplementary Materials

The following supporting information can be downloaded at: https://www.mdpi.com/article/10.3390/nu16152395/s1, Table S1: PRISMA 2020 Checklist.
